# Galectin fingerprinting in naso-sinusal diseases

**DOI:** 10.3892/or.2014.3213

**Published:** 2014-05-23

**Authors:** ANAËLLE DURAY, THIBAULT DE MAESSCHALCK, CHRISTINE DECAESTECKER, MYRIAM REMMELINK, GILBERT CHANTRAIN, JENNIFER NEIVEYANS, MIHAELA HOROI, XAVIER LEROY, HANS-JOACHIM GABIUS, SVEN SAUSSEZ

**Affiliations:** 1 Laboratory of Anatomy, Faculty of Medicine and Pharmacy, University of Mons, Mons, Belgium; 2 Department of Oto-Rhino-Laryngology, CHU Saint-Pierre, Université Libre de Bruxelles, Brussels, Belgium; 3 Laboratory of Image, Signal Processing and Acoustics (LISA), Ecole Polytechnique de Bruxelles, Université Libre de Bruxelles, Brussels, Belgium; 4 Department of Pathology, Hôpital Erasme, Université Libre de Bruxelles, Brussels, Belgium; 5 Department of Pathology, Faculty of Medicine, Hôpital Claude Huriez and Centre de Biologie-Pathologie, CHRU, Lille, France; 6 Institute of Physiological Chemistry, Faculty of Veterinary Medicine, Ludwig Maximilian University of Munich, Munich, Germany

**Keywords:** galectin, immunohistochemical, papilloma, polyposis, rhinosinusitis, adenocarcinoma

## Abstract

Galectins, a family of endogenous lectins, are multifunctional effectors that act at various sites and can be used in immunohistochemical localization studies of diseased states. Since they form a potentially cooperative and antagonistic network, we tested the hypothesis that histopathological fingerprinting of galectins could refine the molecular understanding of naso-sinusal pathologies. Using non-cross-reactive antibodies against galectin-1, -3, -4, -7, -8 and -9, we characterized the galectin profiles in chronic rhinosinusitis, nasal polyposis, inverted papillomas and squamous cell carcinomas. The expression, signal location and quantitative parameters describing the percentage of positive cells and labeling intensity were assessed for various cases. We discovered that inverted papillomas showed a distinct galectin immunohistochemical profile. Indeed, epithelial overexpression of galectin-3 (P=0.0002), galectin-4 (P<10^−6^), galectin-7 (P<10^−6^) and galectin-9 (P<10^−6^) was observed in inverted papillomas compared to non-malignant diseases. Regarding carcinomas, we observed increased expression of galectin-9 (P<10^−6^) in epithelial cells compared to non-tumor pathologies. Our results suggest that galectin-3, -4, -7 and -9 could be involved in the biology of inverted papillomas. In addition, we observed that the expression of galectin in naso-sinusal diseases seems to be affected by tumor progression and not inflammatory or allergic phenomena.

## Introduction

The increasing awareness of the ability of glycans to store biological information (coding by sugars) has directed research efforts toward endogenous lectins (1). In immunology and tumor biology, the interplay between glycan remodeling and lectin expression constitutes a potent molecular switch for cell adhesion and growth regulation, which affects, for example, activated T (effector) cells or carcinoma cells after the reconstitution of tumor suppressor p16^INK4a^ (2–8). Members of the family of galectins (Gals), which share the β-sandwich fold and reactivity to galactosides, play a prominent role in this respect (9,10). Of note, they are multifunctional proteins with an activity spectrum beyond decoding cell surface glycans (11,12). As our previous studies on galectin localization in head and neck tumors exemplarily demonstrated, they can be detected in the cytoplasm and nucleus, with shifts in localization occurring during progression (13–18). Gals target distinct counter receptors that are located on the cell surface and in the extracellular matrix, such as bcl-2, using Gal-3 and -7 as part of their functionality (12,19). Additionally, our studies have also documented the presence of a network of these effectors. As a consequence, the study design should progress from monitoring individual members of this class to testing a panel of non-cross-reactive antibodies. Database mining has previously revealed sequence divergence at the promoter level and variations in the gene copy-number among Gals (20); however, the immunohistochemical fingerprinting approach will provide insights into the regulation of individual family members. In the present study, we applied this technique to comparatively analyze the expression of Gal-1, -3, -4, -7, -8 and -9, the entire group of human lectins, in naso-sinusal pathologies. The following diseases were examined: chronic rhinosinusitis (CRS), nasal polyposis, inverted papillomas and squamous cell carcinomas. The origin of manifestation can be either inflammatory, such as in CRS and naso-sinusal polyposis or tumoral (inverted papillomas and squamous cell carcinomas). For most cases, the etiology and pathogenesis are not yet completely known. Therefore, establishing an early diagnosis is difficult, particularly since symptoms are not specific. CRS affects ~15% of the population and is defined as inflammation of one or more of the paranasal sinuses that lasts >12 weeks (21,22). In general, CRS is divided into three categories: CRS with nasal polyps, CRS without nasal polyps, and allergic fungal rhinosinusitis (23). A fourth group, eosinophilic CRS, that is characterized by the presence of a high number of activated eosinophils in the mucosa was also proposed (24,25). The latter group is often associated with a more severe disease and diminished surgical success (26). Naso-sinusal polyposis is characterized by inflammatory outgrowths of paranasal sinus mucosa caused by chronic mucosal inflammation, typically arising from the middle meatus and ethmoid region. It is a common disease affecting up to 4% of the general population (27,28). In the present study, we focused specifically on non-allergic nasal polyps and allergic nasal polyps, which present inflammatory mediators, eosinophils and sensitivity to allergens (29). Typically, patients with CRS or naso-sinusal polyposis have nasal obstruction, anosmia, rhinorrhea and facial pain (30). Inverted papillomas are sinonasal lesions primarily on the lateral nasal wall that are characterized by recurrence potential and the propensity for malignancy (31,32). The term inverted papilloma describes the reversal of epithelial proliferation, which is endophytic and does not affect the basement membrane (33). Epstein-Barr virus (EBV) or human papillomavirus (HPV) are implicated in its pathogenesis (34). Finally, squamous cell carcinoma stems from the epithelium of the respiratory mucosa of the nasal cavity and paranasal sinuses. It is a rare malignancy, representing <1% of malignant tumors and ~3% of malignancies affecting the head and neck. Early diagnosis is difficult because symptoms and signs are not specific but are similar to those of chronic sinusitis, allergic reactions and nasal polyps, i.e., symptoms caused by nasal obstruction (35).

Therefore, our disease panel was suited to address Gal regulation, with potential implications for the diagnosis of 90 cases. We assessed the expression, localization and semi-quantitative parameters, such as signal intensity, percentage of stained areas and percentage of positive cells, for each case.

## Materials and methods

### Patient characteristics

Specimens were surgically removed from 90 patients with naso-sinusal pathologies and studied. The specimens included 29 chronic rhinosinusitises, 26 nasal polyps, 29 inverted papillomas and 6 squamous cell carcinomas (see Table I for clinical data). The specimens were obtained by a retrospective compilation of the records from the Department of Pathology at the Hôpital Claude Huriez (Lille, France), the CHU Saint-Pierre (Brussels, Belgium) and the Centre Epicura (Baudour, Belgium). The institutional review boards of these hospitals approved the study (AK/09-09-47/3805AD). Haematoxylin and eosin-stained (H&E)sections from the 90 cases were routinely examined by two pathologists to confirm the diagnosis.

### Antibodies

Human Gal-1, -3, -4, -7, -8 and -9 were produced in bacteria, purified to homogeneity, as confirmed by one-dimensional and two-dimensional gel electrophoreses, gel filtration, and mass spectrometry, and used as antigens to develop polyclonal antibodies in rabbits (36–40). The resulting IgG fractions were rigorously checked for cross-reactivity among the lectin family, with systematic testing of human Gal-1, -2, -3, -4, -7, -8 and -9 by western blot analysis and enzyme-linked immunosorbent assay. Chromatographic affinity depletion was performed on galectin-presenting Sepharose 4B in the case of positivity, followed by quality control to ascertain the elimination of cross-reactivity, as previously described (41–43).

### Immunohistochemistry

All tumor samples were fixed in 4% buffered formaldehyde for 24 h, dehydrated and embedded in paraffin. Immunohistochemistry was performed on 5-μm thick sections mounted on silane-coated glass slides (18). Before starting the immunohistochemistry protocol, deparaffinized tissue sections were placed in 0.01 M citrate buffer (pH 6.0) and briefly pre-treated in a 900 W microwave for 2×5 min. The sections were then incubated in a solution containing 0.06% H_2_O_2_ for 5 min to block endogenous peroxidase activity, rinsed in phosphate-buffered saline (PBS; 0.04 M Na_2_HPO_4_, 0.01 M KH_2_PO_4_ and 0.12 M NaCl, pH 7.4) and successively exposed to solutions containing avidin (0.1 mg/ml in PBS) and biotin (0.1 mg/ml in PBS), respectively, for 5 min each to prevent false-positive staining reactions due to the presence of endogenous biotin. After thorough washing with PBS, the sections were incubated for 15 min in a solution containing 0.5% casein in PBS and sequentially exposed to solutions containing the following proteins at room temperature: i) the specific primary antibody; ii) the corresponding biotinylated secondary antibody (polyclonal goat anti-rabbit IgG); and iii) the avidin-biotin-peroxidase complex (ABC). The samples were thoroughly washed between incubation steps to remove unbound proteins. The antigen-dependent presence of the peroxidase complex in the sections was visualized by incubating diaminobenzidine and H_2_O_2_ with the chromogenic substrates. After rinsing, the sections were counterstained with Luxol Fast Blue and mounted in synthetic medium. To exclude antigen-independent staining in the control samples, incubation steps with the primary/secondary antibodies were omitted from the protocol. In all instances, these controls were negative. The biotinylated secondary antibodies and ABC kit were obtained from DakoCytomation (Glostrup, Denmark).

### Semi-quantitative analysis

For each specimen (15 microscopic fields), we focused our analysis on the epithelial and stromal components. The signal intensity of the immunoreactivity (mean intensity, MI) was scored as either 0 (negative), 1 (weak), 2 (moderate) or 3 (strong), and the population of positive cells (labeling index, LI) was expressed as the percentage of immunopositive cells. The quick score (QS) was calculated by multiplying the score for the reactivity intensity by the percentage of immunopositive cells.

### Data analysis

Independent groups of quantitative data were compared with the non-parametric Kruskall-Wallis test (more than two groups). In the case of significant results, post-hoc tests (Dunn procedure) were used to compare pairs of groups (to avoid multiple comparison effects). A P-value of <0.05 was considered to indicate a statistically significant result.

## Results

The first observation was on the range of galectin immunohistochemical positivity. In most cases, the application of the 6 anti-Gal antibodies revealed the presence of lectins. We decided to describe the galectin fingerprinting of each naso-sinusal disease separately.

**Figure 1:**
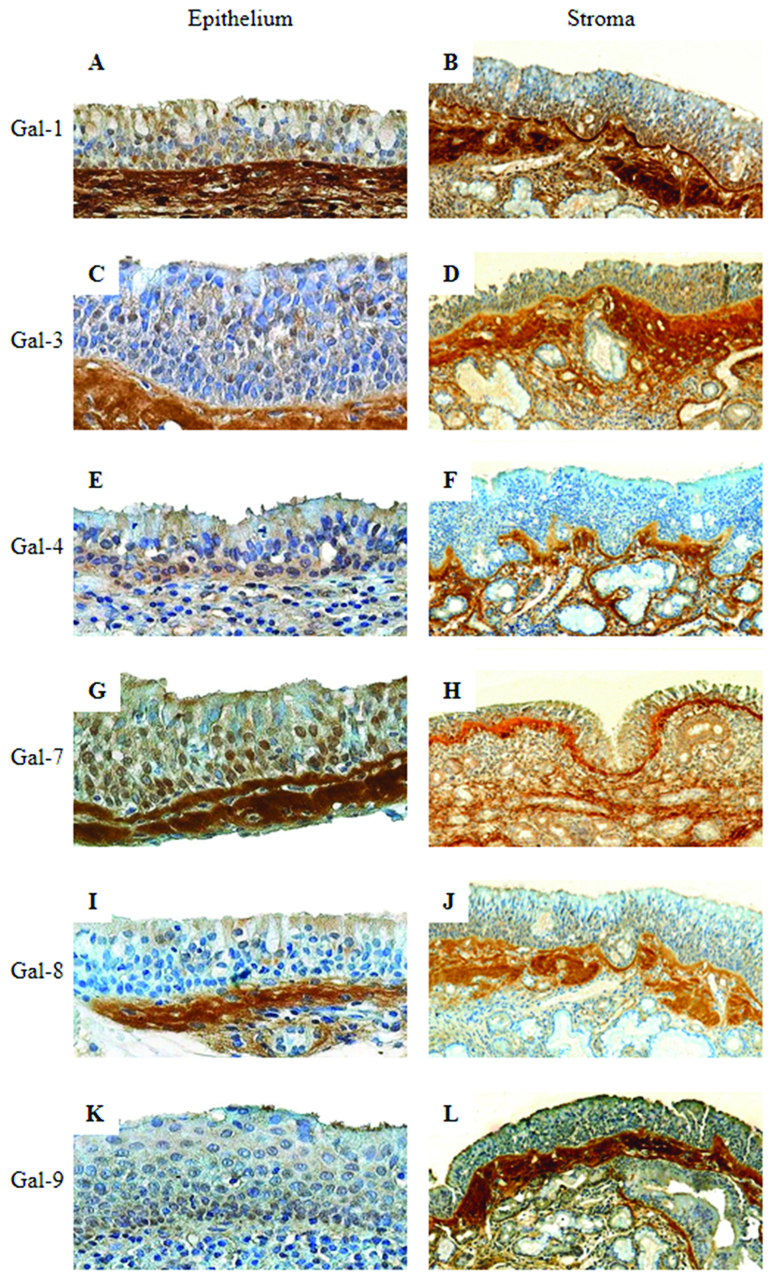
Immunohistochemical expression profiles for galectin-1, galectin-3, galectin-4, galectin-7, galectin-8 and galectin-9 in the epithelium (A, C, E, G, I and K; original magnification, ×400) and stroma (B, D, F, H, J and L; original magnification, ×100) of non-eosinophilic chronic rhinosinusitis.

**Figure 2:**
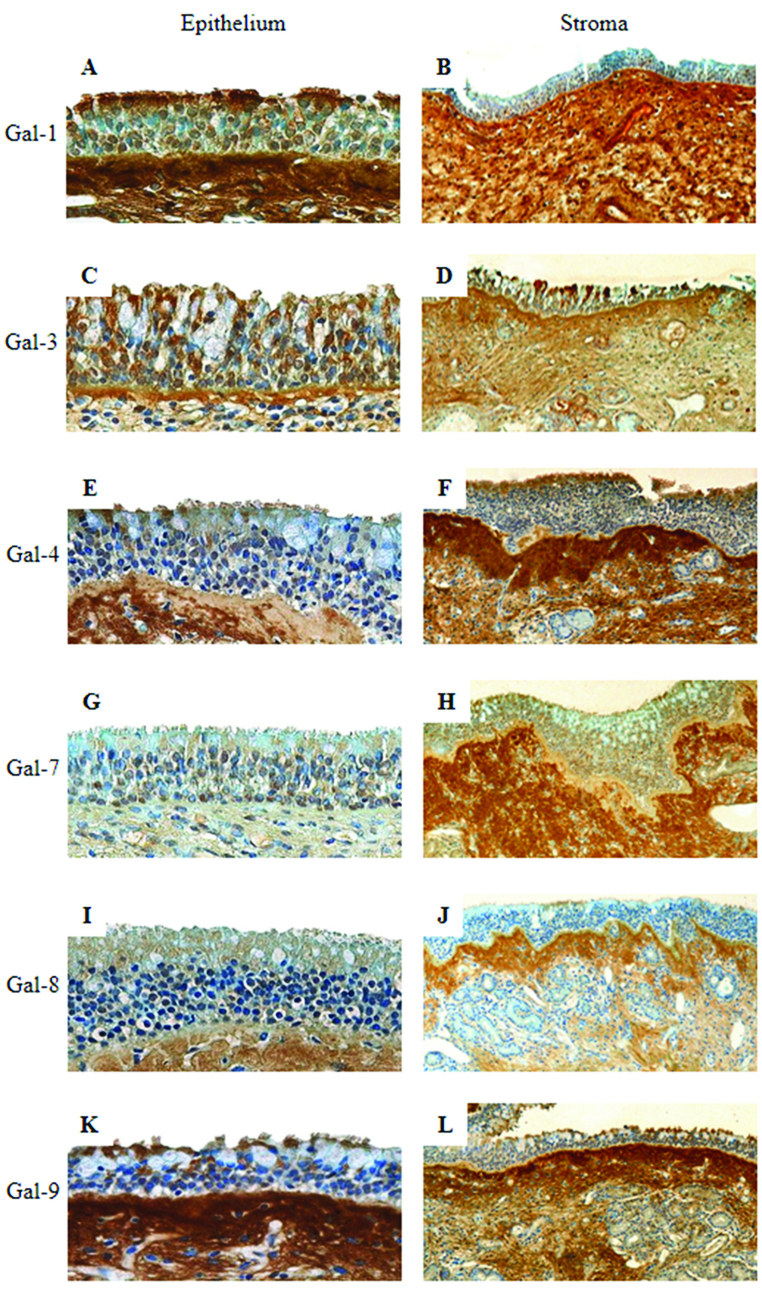
Immunohistochemical expression profiles for galectin-1, galectin-3, galectin-4, galectin-7, galectin-8 and galectin-9 in the epithelium (A, C, E, G, I and K; original magnification, ×400) and stroma (B, D, F, H, J and L; original magnification, ×100) of eosinophilic chronic rhinosinusitis.

All of the 10 non-eosinophilic chronic rhinosinusitis (NECRS) cases showed positive staining for the 6 Gals. Of the cases of eosinophilic chronic rhinosinusitis (ECRS) (19 cases), 100% of the specimens were positive for Gal-1, -3 and -7, 94% were positive for Gal-8 and 89% were positive for Gal-9. The immunostaining was predominately nucleocytoplasmic (Table II). In NECRS, the signal intensity was low except for Gal-7, which was moderately expressed (Fig. 1A, C, E, G, I and K). In fact, the immunohistochemical profiles of ECRS cases differed from the non-eosinophilic cases. In detail, a low intensity was detected for Gal-4 and -8, a low to moderate intensity was recorded for Gal-1, -3 and -9 and a moderate intensity was observed for Gal-7 (Fig. 2A, C, E, G, I and K). Statistical analysis shows a decreased positive area for Gal-3 in NECRS (post-hoc comparison, P=0.02) and Gal-7 in ECRS (post-hoc comparison, P=0.0003) compared to cases with inverted papillomas (IPs, 29 cases) (Fig. 7A and C). Compared to the IPs and squamous cell carcinomas (SCCs, 6 cases), the percentage of Gal-9-immunopositive cells (Fig. 7E) was downregulated in NECRS (post-hoc comparisons, P=0.02 for both) and ECRS (post-hoc comparisons, P=0.0006 and P=0.003, respectively). Concerning the immunohistochemical detection of Gal-1, -4 and -8, no significant difference was observed between CRS and the other lesions (Fig. 7B and D). Therefore, ECRS expressed a low level of Gal-7 and -9, whereas non-eosinophilic lesions expressed a low level of Gal-3 and -9 (Fig. 7A, C and E).

**Figure 3:**
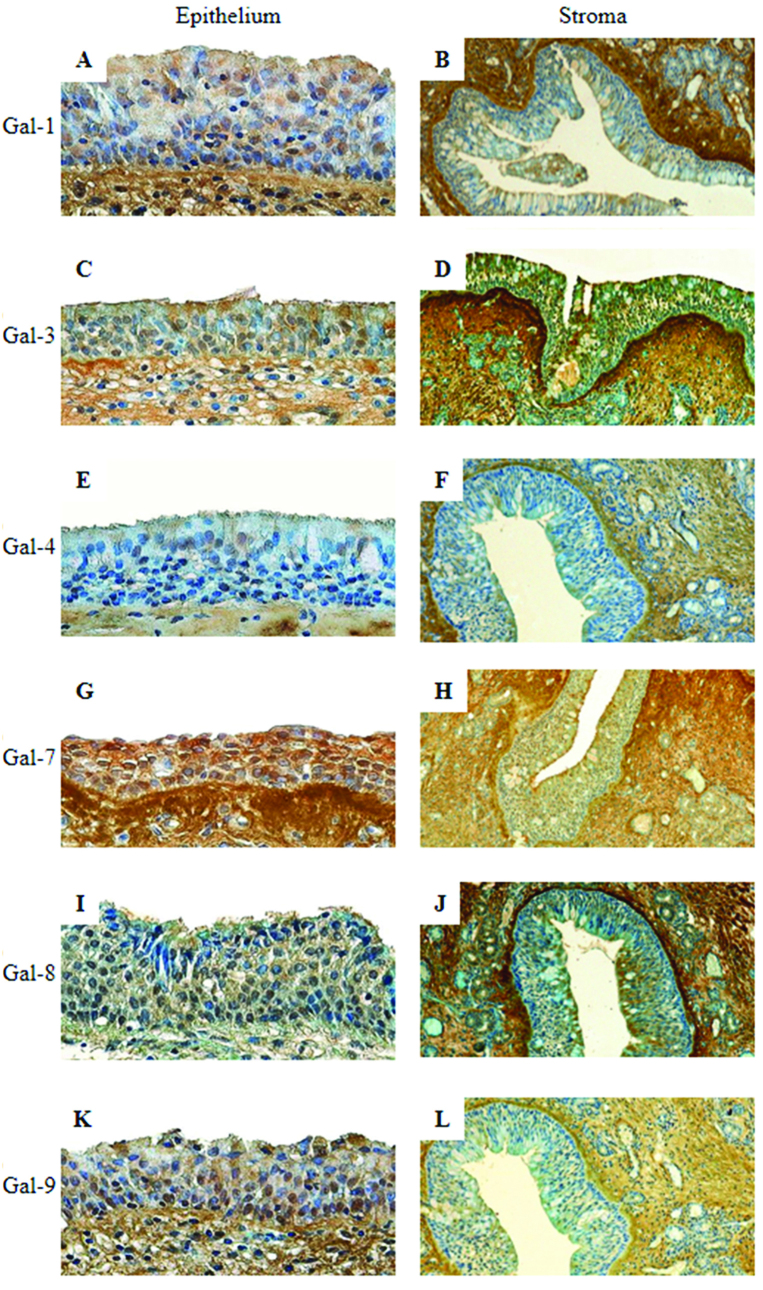
Immunohistochemical expression profiles for galectin-1, galectin-3, galectin-4, galectin-7, galectin-8 and galectin-9 in the epithelium (A, C, E, G, I and K; original magnification, ×400) and stroma (B, D, F, H, J and L; original magnification, ×100) of a non-allergic nasal polyp.

**Figure 4:**
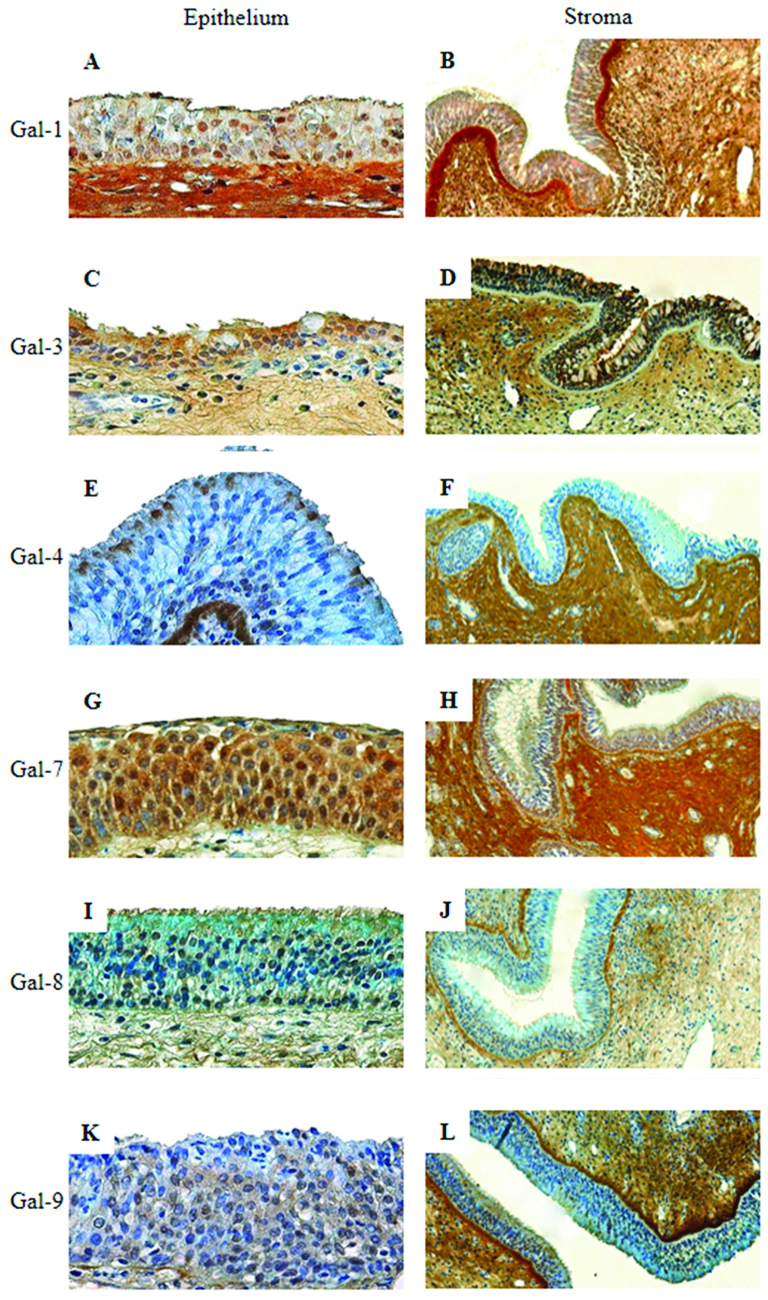
Immunohistochemical expression profiles for galectin-1, galectin-3, galectin-4, galectin-7, galectin-8 and galectin-9 in the epithelium (A, C, E, G, I, K; original magnification, ×400) and stroma (B, D, F, H, J, L; original magnification, ×100) of an allergic nasal polyp.

The polyp group was subdivided into non-allergic nasal polyps (NANPs; 9 cases) and allergic nasal polyps (ANPs; 17 cases). All epithelial cells were moderately positive for Gal-3 and -7 with nucleocytoplasmic distribution (Figs. 3C and G; 4C and G) (Table II). For the other 4 Gals, the percentage of positive cases (i.e., with LI >0%) did not significantly vary according to the type of lesion (NANP vs. ANP), except for Gal-4 (55 vs. 88% of positive cases). These two types of lesions are shown in Fig. 3 and 4, which reveal low to moderate nucleocytoplasmic or cytoplasmic positivity, respectively. As illustrated in Fig. 7B, a lower percentage of Gal-4-immunopositive cells was detected in ANPs and NANPs (post-hoc comparisons, P=0.0004 and P=0.0007, respectively) compared to IPs. Downregulation of Gal-3 and -7 (post-hoc comparisons, P=0.0003 and P=0.000002, respectively) was detected in ANPs compared to IPs (Fig. 7A and C). We also observed a lower percentage of Gal-9-positive cells in ANPs than in IPs or SCCs (post-hoc comparison, P=0.01 and P=0.02, respectively) (Fig. 7E). A statistically significant difference was not observed between the expression of Gal-8 in nasal polyps and the expression of Gal-8 in the other pathologies (Fig. 7D). The immunohistochemical profile of nasal polyps differed with respect to the allergic status of the patients. In fact, the observations show that ANPs were low in Gal-3, -4, -7 and -9, whereas NANPs were only low in Gal-4.

**Figure 5:**
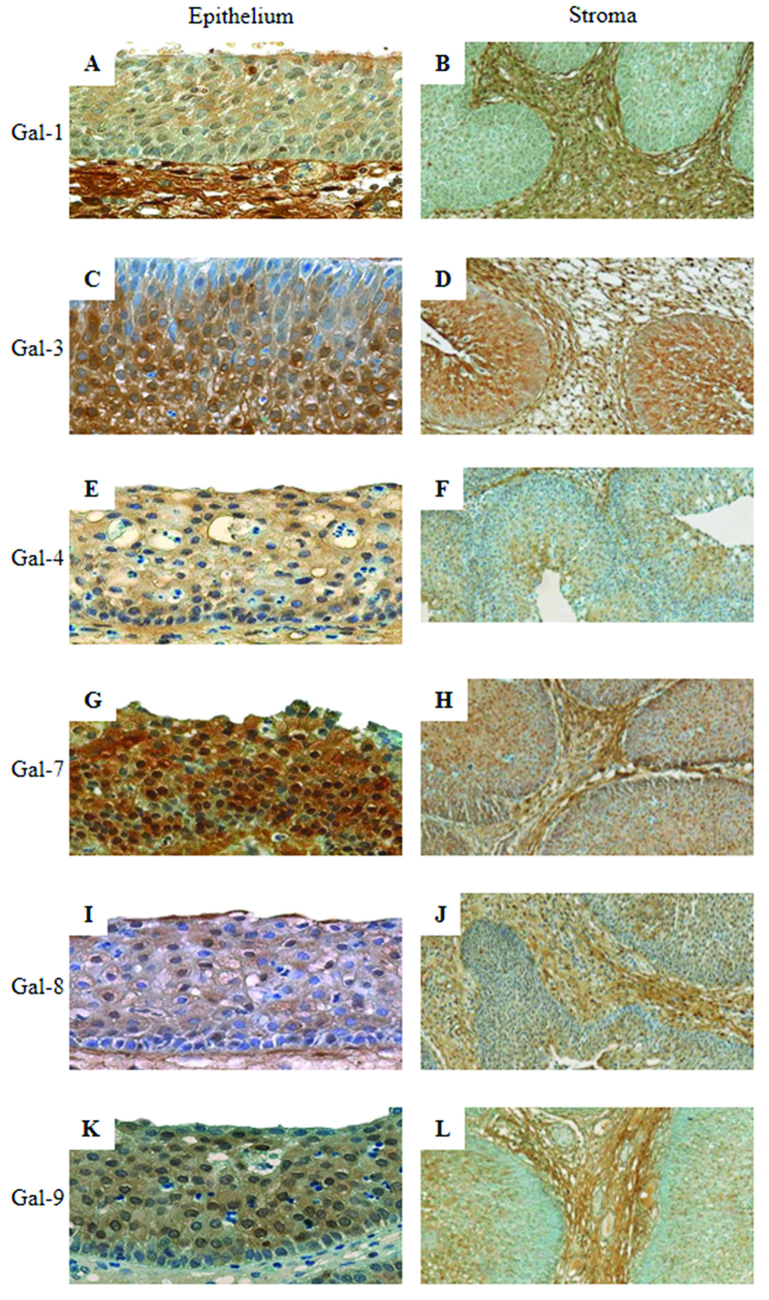
Immunohistochemical expression profiles for galectin-1, galectin-3, galectin-4, galectin-7, galectin-8 and galectin-9 in the epithelium (A, C, E, G, I and K; original magnification, ×400) and stroma (B, D, F, H, J, and L; original magnification, ×100) of an inverted papilloma.

In inverted papillomas (IPs), nucleocytoplasmic immunostaining of Gal-1, -3 and -7 was detected in 100% of epithelial cells. Slightly lower percentages of 96 and 93% were detected for Gal-4 and both Gal-8 and -9, respectively. Galectins were present in both the nuclei and cytoplasm. Only Gal-9 was invariably present in epithelial cells (Table II). As shown in Fig. 5 for epithelial cells, a low to moderate signal was detected for Gal-1, -4 and -8, and a moderate to intense signal was detected for Gal-3, -7 and -9. An analysis of the quantitative data showed an increased percentage of positivity for the following Gals in the epithelium of IPs: Gal-3 compared to ANPs and NECRS (post-hoc comparison, P=0.0003 and P=0.002, respectively) (Fig. 7A), Gal-4 compared to ANPs and NANPs (post-hoc comparison, P=0.0004 and P=0.0007, respectively) (Fig. 7B), Gal-7 compared to ANPs and ECRS (post-hoc comparison, P=0.000002 and P=0.0003, respectively) (Fig. 7C), Gal-8 compared to SCCs (post-hoc comparison, P=0.004) (Fig. 7D) and Gal-9 compared to ANPs, ECRS and NECRS (post-hoc comparison, P=0.01, P=0.0006 and P=0.02, respectively) (Fig. 7E). IPs are thus characterized by the overexpression of Gal-3, -4, -7, -8, -9 but not Gal-1.

**Figure 6:**
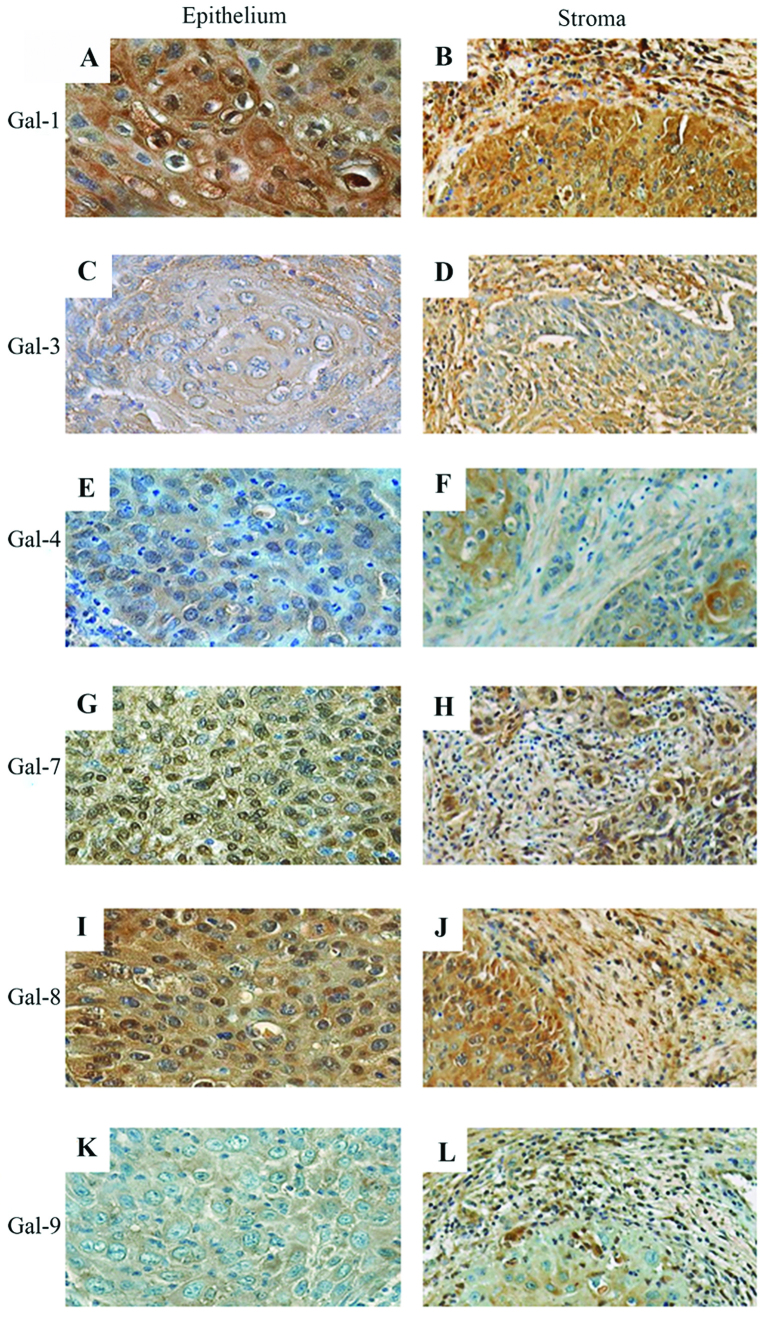
Immunohistochemical expression profiles for galectin-1, galectin-3, galectin-4, galectin-7, galectin-8 and galectin-9 in the epithelium (A, C, E, G, I and K; original magnification, ×400) and stroma (B, D, F, H, J and L; magnification, ×200) of squamous cell carcinoma.

**Figure 7:**
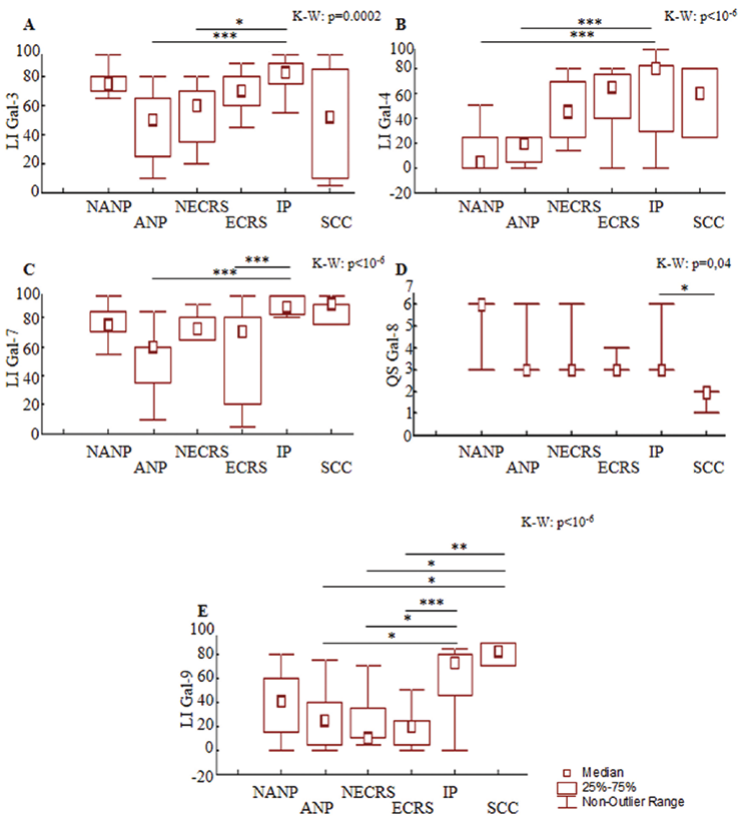
Results of the semi-quantitative percentage of immunopositive cells for galectin-3, galectin-4, galectin-7 or galectin-9 [labeling index (LI)] (A, B, C and E) and the quick score (QS) for galectin-8 (i.e., multiplication of the signal intensity score by the LI score) (D) for 10 cases of non-eosinophilic chronic rhinosinusitis (NECRS), 19 cases of eosinophilic chronic rhinosinusitis (ECRS), 9 cases of non-allergic nasal polyps (NANPs), 17 cases of allergic nasal polyps (ANPs), 29 cases of inverted papillomas (IPs) and 6 cases of squamous cell carcinomas (SCCs). Significant post-hoc comparison results are indicated by the lines (indicating the pairs of groups being compared) (^*^P<0.05, ^**^P<0.01, ^***^P<0.001). The result of the Kruskall-Wallis test is indicated in the top-left corner of each frame.

The epithelial cells in naso-sinusal SCCs were positive for all of the galectins, except Gal-8 (83%). The localization depends on the type of lectin. The nucleocytoplasmic or cytoplasmic expression pattern contrasted with the exclusively cytoplasmic positivity observed for Gal-1 and -9 (Fig. 6; Table II). The signal intensity detected was moderate for Gal-1 and low to moderate for the other Gals, as shown in Fig. 6A higher percentage of Gal-9-positive cells was detected in SCCs compared to ANPs, NECRS and ECRS (post-hoc comparisons, P=0.02, P=0.02 and P=0.003, respectively) (Fig. 7E). In contrast, a lower Gal-8 quick score was determined for SCCs compared to IPs (post-hoc comparison, P=0.04; Fig. 7D). No significant difference was found for the presence of Gal-1, -3, -4 and -7 (Fig. 7A–C). Thus, the immunohistochemical profile of nasal carcinomas appears to be characterized by a high-level of Gal-9 and a low-level of Gal-8.

In the 90 cases studied, almost all of the stromal cells exhibited staining for the 6 galectins. Similar immunohistochemical profiles were revealed for NECRS and ANPs, i.e., low to moderate positivity (Figs. 1B, D, F, H, J and L; 4B, D, F, H, J and L). Gal-1 and -4 were moderately positive in ECRS, NANPs and IPs (Figs. 2B and F; 3B and F; 5B and F). The staining intensity of Gal-3, -7, -8 and -9 was either low, low to moderate or moderate depending on the type of lesion, i.e., ECRS, NANPs and IPs (Figs. 2D, H, J and L; 3D, H, J and L; 5D, H, J and L). In contrast, galectin expression was relatively low in the SCCs studied (Fig. 6B, D, F, H, J and L).

## Discussion

Galectin expression is often studied using immunohistochemistry, which focuses on individual family members. The accumulating evidence of this work suggests that effectors can establish a network that warrants combined analysis. The present study explored the characteristic signatures of galectin in several naso-sinusal pathologies. Previous studies of our laboratory that were dedicated to head and neck carcinomas revealed an association between the presence of Gal-1, -3 and -7 and the progression to malignancy, the inverse shifts between nuclear and cytoplasmic localization, and the upregulation of Gal-8 in hypopharyngeal and laryngeal squamous cell carcinomas. These observations contrast with the downregulation that is often encountered in other tumor types, thereby strongly indicating an intriguing difference among galectins (14,17,44). In the present study on a large series of salivary gland tumors, galectin fingerprinting provided a new, significant tool for the differential diagnosis of mucoepidermoid and acinic cell carcinomas. In fact, we observed a unique profile that included the cytoplasmic localization of Gal-1, -3, -7 and -8 in the intermediate cells of mucoepidermoid carcinomas and the absence of Gal-7 expression in acinic cell carcinomas (18). To pursue these observations, the following methodological approach was used in this study: i) monitoring of the expression of 6 galectins in parallel using immunohistochemistry; ii) determination of their localization profiles; and iii) assessment of the semi-quantitative expression parameters. In detail, we compared the parameters for Gal-1 and Gal-7 (proto-type), Gal-3 (chimera-type), and Gal-8 and -9 (tandem-repeat-type) to provide several key insights, such as the non-uniform regulation of individual homologous proteins. The study also further verifies the nucleocytoplasmic existence of galectins, which is a common location for these proteins (44,45).

We determined that allergies do not seem to affect galectin presence. Our results showed no significant difference between NANPs and ANPs for all galectins studied, which is consistent with previous results obtained for Gal-1 and -3 (27). Indeed, they showed that the expression of galectin-1 was significantly higher in nasal polyps than in middle turbinates. They also detected the increased expression of galectin-3 in nasal polyps compared to middle and inferior turbinates. However, they showed no relationship between the allergic status of the patient and the expression of these galectins (27). Galectin-3, formerly identified as IgE-binding protein, is also suspected to play a role in allergy pathways (46). It is remarkable that the allergic status of the patient had no apparent effect on the expression of this galectin. Another study by Sena and colleagues (47) used an immunohistochemical and molecular approach to investigate the expression of Gal-1 and glucocorticoid-regulated protein Annexin 1 (ANXA1). They observed the upregulation of ANXA1 and the downregulation of Gal-1 in polypoid tissue compared to normal mucosa. The authors suggested that this observation could be associated with a specific mechanism in NPs. Regarding the presence of Gals in CRS, no significant difference between NECRS and ECRS was observed. Although Gal-9 is considered a chemoattractant for eosinophils (48), its expression is not modulated by eosinophils. Notably, nuclear Gal-9 can physically interact with NF-IL6 (C/EBP-β), a transcription factor of the basic leucine zipper family, in monocytic cells (49). If we consider all inflammatory conditions, no significant difference was observed between nasal polyposis and chronic rhinosinusitis. Sinonasal polyposis is the final stage of chronic rhinosinusitis, and the expression of galectin does not seem to be affected by the severity of the inflammatory process.

In the present study, we describe the significant upregulation and downregulation of Gals in naso-sinusal tumoral progression. An increased number of epithelial cells positive for Gal-4, -7 and -9 was detected in inverted papillomas and carcinomas compared to non-malignant disease. The anti-apoptotic activity of intracellular Gal-3 may contribute to tumor cell survival. Concerning squamous cell carcinoma, a decrease in nuclear but not cytoplasmic Gal-3 was observed. The overexpression of galectin-9, which is known to suppress the adhesion of tumor cells to the extracellular matrix and vascular endothelium, could be advantageous for the treatment of squamous cell carcinomas because it limits the formation of metastases (50). Exosomal packaging, which has been shown to induce apoptosis of EBV-specific CD4^+^ cells in nasopharyngeal carcinomas (51–53), can describe a route for intracellular Gal-9 to become an extracellular effector. Finally, a decrease in galectin-8 in carcinomas, but not IPs, was shown. Hadari and colleagues (54) showed that galectin-8, which is involved in anoikis, was able to interact with integrins and, therefore, inhibit adhesion to the extracellular matrix and promote apoptosis. Based on these data, a greater amount of galectin-8 in IPs suggests that galectin-8 is involved in the prevention of carcinogenesis by inhibiting cell-extracellular matrix interactions.

At this stage, our analysis documents semi-quantitative changes in the cellular expression of Gals. These results suggest a functional correlation by the recognition of distinct counter receptor(s). Methodologically, the application of labeled Gals is an approach to mapping the location of accessible binding sites, which has previously been demonstrated (55,56). Recently, prognostic information was delineated from intracellular Gal-3 reactivity in colon cancer (57). Using the presented map of protein localization, it would be informative to study the reactivity profiles using endogenous lectins as probes. Investigation of galectin-specific signaling pathways in inverted papillomas and carcinomas in future studies are warranted.

## Figures and Tables

**Table I:** Clinical data.

Characteristics	CRS (n=29)	Nasal polyps (n=26)	IPs (n=29)	Carcinomas (n=6)
Age (years)				
Mean	37	43	60	72
Range	18–63	10–74	27–84	53–86
Gender				
Male	22	18	20	6
Female	7	8	9	0
Treatment				
Surgery	29	26	29	6
Histology				
Non-eosinophilic	10	-	-	-
Eosinophilic	19	-	-	-
Non-allergic	-	9	-	-
Allergic	-	17	-	-
Squamous cell	-	-	-	6
TNM stage				
I	-	-	-	0
II	-	-	-	0
III	-	-	-	0
IV	-	-	-	6

CRS, chronic rhinosinusitis; IPs, inverted papillomas.

**Table II:** Intracellular localization of galectin-1, -3, -4, -7, -8 and -9.^a^

	Galectin-3 localization			Galectin-3 localization			Galectin-4 localization			Galectin-7 localization			Galectin-8 localization			Galectin-9 localization		
						
Type of tissue	C n/total (%)	N n/total (%)	C + N n/total (%)	C n/total (%)	N n/total (%)	C + N n/total (%)	C n/total (%)	N n/total (%)	C + N n/total (%)	C n/total (%)	N n/total (%)	C + N n/total (%)	C n/total (%)	N n/total (%)	C + N n/total (%)	C n/total (%)	N n/total (%)	C + N n/total (%)
Non-eosinophilic chronic rhinosinusitis (n=10)	0/10 (0)	0/10 (0)	10/10 (100)	0/10 (0)	0/10 (0)	10/10 (100)	2/10 (20)	0/10 (0)	8/10 (80)	0/10 (0)	0/10 (0)	10/10 (100)	0/10 (0)	0/10 (0)	10/10 (100)	2/10 (20)	0/10 (0)	8/10 (80)
Eosinophilic chronic rhinosinusitis (n=19)	0/19 (0)	0/19 (0)	19/19 (100)	0/19 (0)	0/19 (0)	19/19 (100)	2/17 (12)	0/17 (0)	15/17 (88)	0/19 (0)	0/19 (0)	19/19 (100)	0/17 (0)	0/17 (0)	17/17 (100)	11/17 (65)	0/17 (0)	6/17 (35)
Non-allergic nasal polyps (n=9)	2/8 (25)	0/8 (0)	6/8 (75)	0/9 (0)	0/9 (0)	9/9 (100)	0/5 (0)	0/5 (0)	5/5 (100)	0/9 (0)	0/9 (0)	9/9 (100)	0/9 (0)	0/9 (0)	9/9 (100)	0/8 (0)	0/8 (0)	8/8 (100)
Allergic nasal polyps (n=17)	2/17 (12)	0/17 (0)	15/17 (88)	0/17 (0)	0/17 (0)	17/17 (100)	5/15 (33)	0/15 (0)	10/15 (67)	0/17 (0)	0/17 (0)	17/17 (100)	2/16 (12)	0/16 (0)	14/16 (88)	3/16 (19)	0/16 (0)	13/16 (81)
Inverted papillomas (n=29)	2/28 (7)	0/28 (0)	26/28 (93)	1/24 (4)	0/24 (0)	23/24 (96)	18/27 (67)	0/27 (0)	9/27 (33)	0/27 (0)	0/27 (0)	27/27 (100)	4/27 (15)	0/27 (0)	23/27 (85)	6/27 (22)	2/27 (8)	19/27 (70)
Squamous cell carcinomas (n=6)	6/6 (100)	0/6 (0)	0/6 (0)	5/6 (83)	0/6 (0)	1/6 (16)	2/6 (33)	0/6 (0)	4/6 (67)	2/6 (33)	0/6 (0)	4/6 (67)	2/5 (40)	0/5 (0)	3/5 (60)	6/6 (100)	0/6 (0)	0/6 (0)

a Separated into cytoplasmic (C) and/or nuclear (N) signals. The number of cases and the percentage of immunopositive cases relative to the intracellular localization (in parentheses) are provided for each type of tissue.

## Abbreviations

ANP: allergic nasal polyp
NANP: non-allergic nasal polyp
CRS: chronic rhinosinusitis
ECRS: eosinophilic chronic rhinosinusitis
NECRS: non-eosinophilic chronic rhinosinusitis
IP: inverted papilloma
SCC: squamous cell carcinoma
